# Supplementary material for: Methods to establish a Pregnancy Register in the QResearch Database
Source: Commun Med (Lond). 2025 Dec 23;5:528. doi: 10.1038/s43856-025-01217-7 (PMC12727747; doi:10.1038/s43856-025-01217-7)
Supplement: Supplementary file 2 — Supplementary Information [file 43856_2025_1217_MOESM2_ESM.pdf]

Supplementary Tables

Supplementary Table 1 – Pregnancy outcomes by identifying data source (30 December 2020 – 30 September 2022)

	Deliveries (livebirth or stillbirth)	Pregnancy loss	All pregnancies
Number of pregnancies (row %)	232,673 (83.4%)	46,354 (16.6%)	279,027
Origin of delivery/loss record, N (column %)			
HES Maternity	229,921 (98.8%)	27 (0.1%)	229,948 (82.4%)
HES Admissions	-	9,348 (20.2%)	9,348 (3.4%)
HES Procedures	-	10,038 (21.7%)	10,038 (3.6%)
GP	2,752 (1.2%)	26,941 (58.1%)	29,693 (10.6%)

Supplementary Figures

Supplementary Figure 1: Phase 2 – Episode Identification

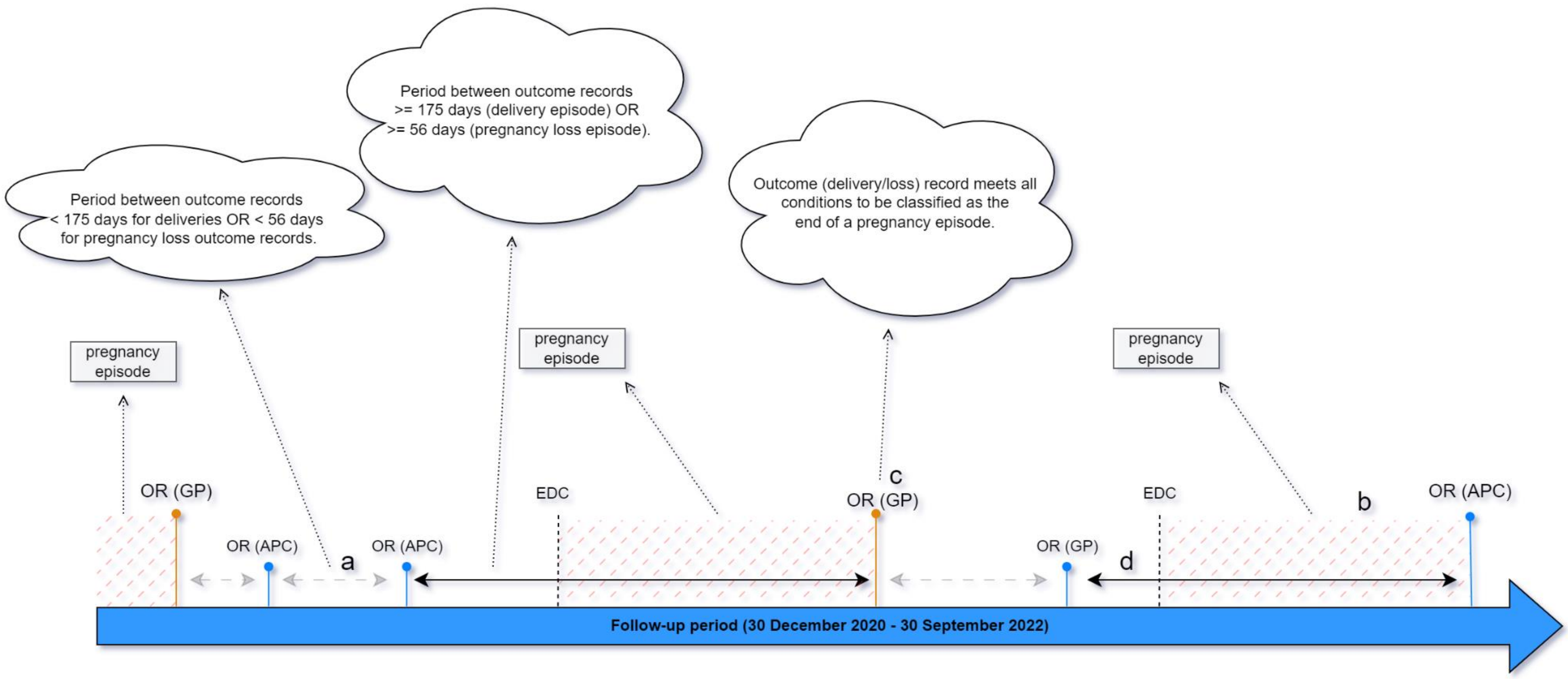

Legend: Diagram displaying criteria used to identify qualifying outcome records (OR) from all datasets (excl. HES Maternity). **a** Period between outcome records is below minimum period required for creation of pregnancy episode – grey dashed line with arrows. **b** Pregnancy episode derived from qualifying dataset (excl. HES Maternity). **c** Pregnancy record derived from GP dataset. **d** Period between outcome records is equal to or exceeds minimum period required for creation of pregnancy episode (delivery/loss) – solid black line with arrows. black dotted line = EDC (Estimated Date of Conception). Blue line =

outcome record from HES Admissions. Orange line = outcome record from primary care. Red dashed lines = period between conception and pregnancy outcome. Large blue arrow = duration of study period.

Supplementary Figure 2: Phase 3 – Episode Identification

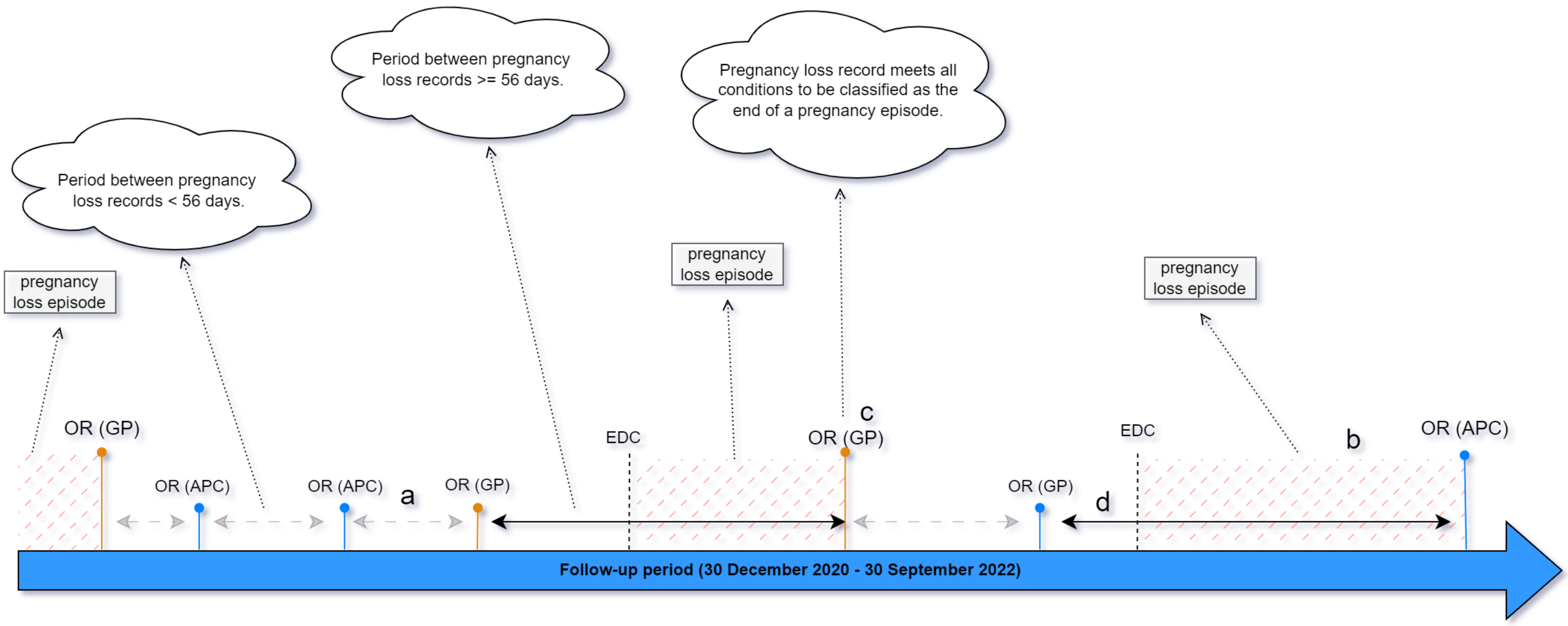

Legend: Diagram displaying criteria used to identify qualifying pregnancy loss outcome records (OR) from all datasets. **a** Period between pregnancy loss records is below minimum period required for creation of loss episode – dashed grey line with arrows. **b** Pregnancy loss episode derived from qualifying

dataset (excl. HES Maternity). **c** Pregnancy record derived from GP database. **d** Period between outcome records is equal to or exceeds minimum period required for creation of pregnancy loss episode – solid black line with arrows. black dotted line = EDC (Estimated Date of Conception). Blue line = outcome record from HES Admissions. Orange line = outcome record from primary care. Red dashed lines = period between conception and pregnancy outcome. Large blue arrow = duration of study period.

Supplementary Figure 3: Venn diagram – Evidence of linking delivery records (n=232,673)* across data sources (same day as delivery date) – 30 December 2020 to 30 September 2022.

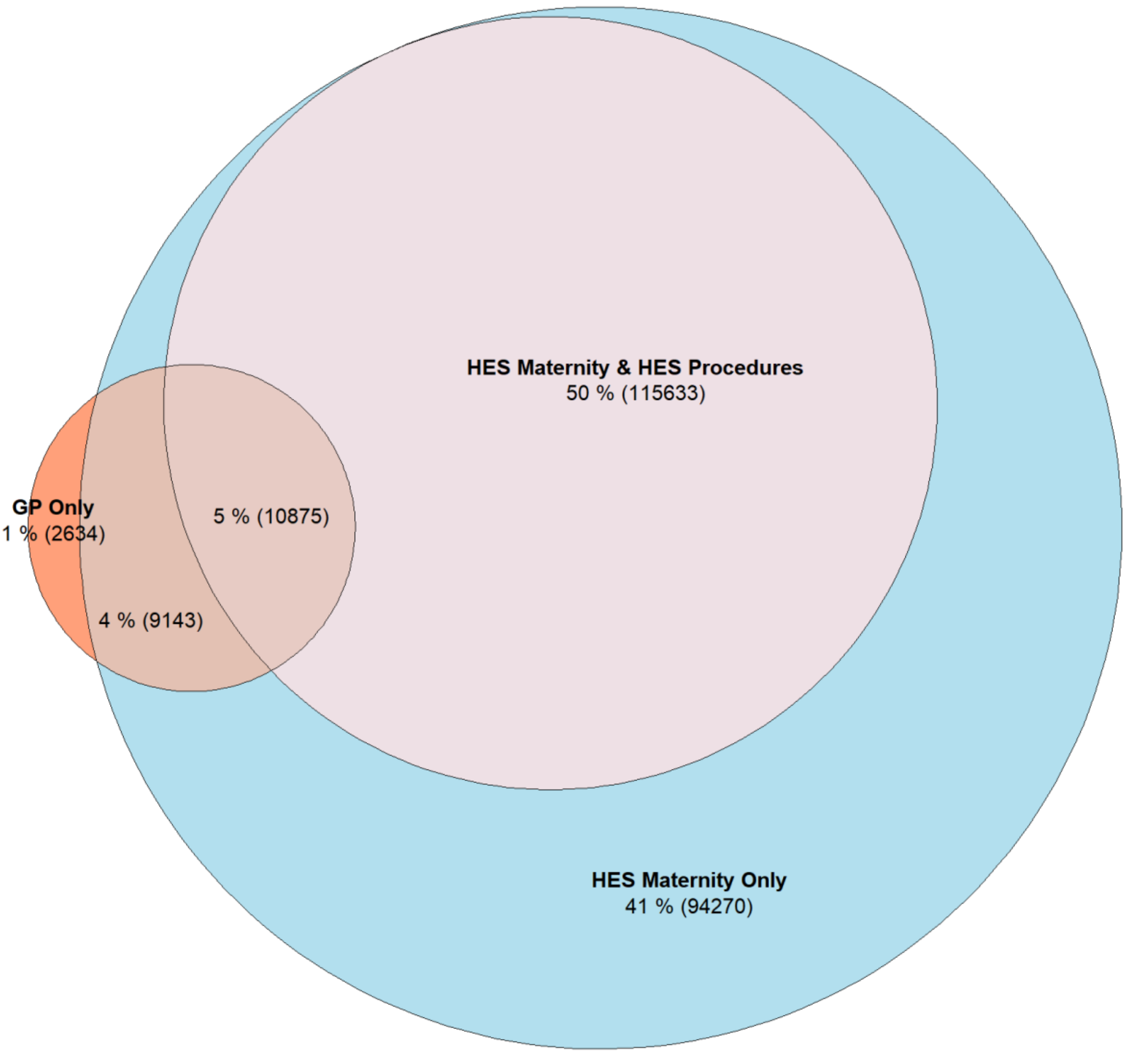

Legend: *Pregnancies suppressed by the Eulerr package due to low counts or omitted because of space restrictions: GP & HES Procedures (n=118). Pink circle = HES Procedures; blue circle = HES Maternity; orange circle = GP. Overlapping areas contain pregnancies with linking records in 1 or more datasets.

Supplementary Figure 4: Venn diagram – Evidence of linking delivery records (n=232,673)* across data sources (±7 days of delivery date) – 30 December 2020 to 30 September 2022.

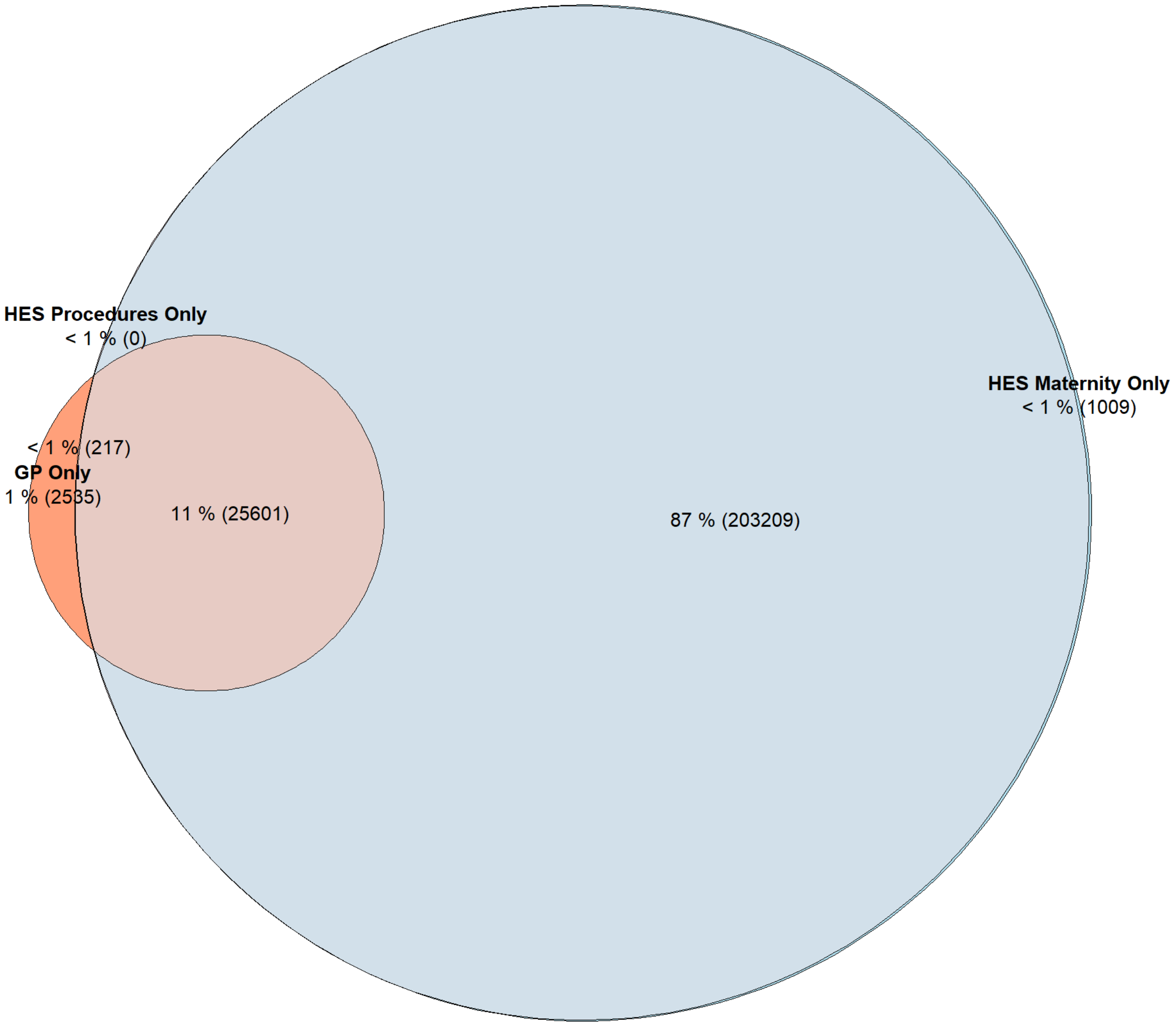

Legend: *Pregnancies suppressed by the Eulerr package due to low counts or omitted because of space restrictions: GP & HES Maternity (n=102). Pink circle = HES Procedures; blue circle = HES Maternity; orange circle = GP. Overlapping areas contain pregnancies with linking records in 1 or more datasets.

Supplementary Figure 5: Venn diagram – Evidence of linking delivery records (n=153,064)* across data sources (same day as delivery date) – 30 December 2020 to 4 February 2022.

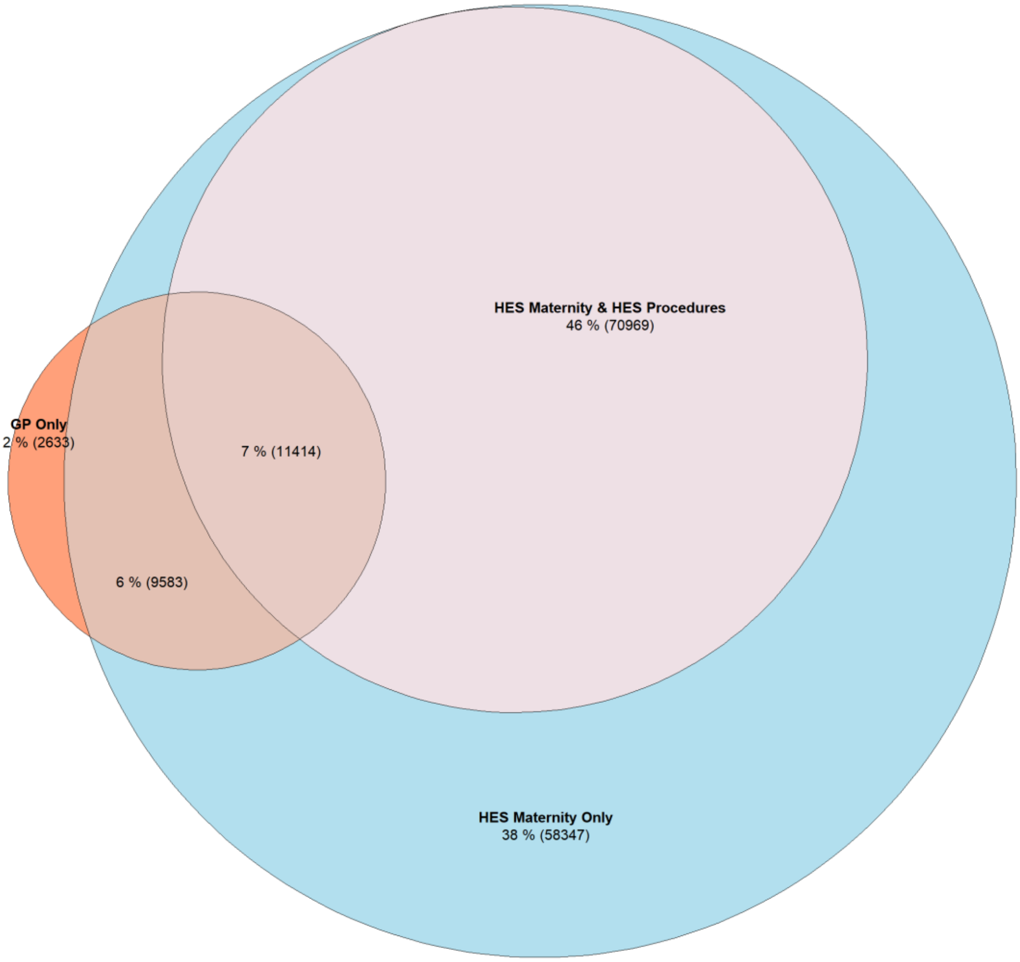

Legend: *Pregnancies suppressed by the Eulerr package due to low counts or omitted because of space restrictions: GP & HES Procedures (n=118). Pink circle = HES Procedures; blue circle = HES Maternity; orange circle = GP. Overlapping areas contain pregnancies with linking records in 1 or more datasets.

Supplementary Figure 6: Venn diagram - evidence of linking delivery records across (n=153,064)* data sources (±2 days of delivery date) – 30 December 2020 to 4 February 2022.

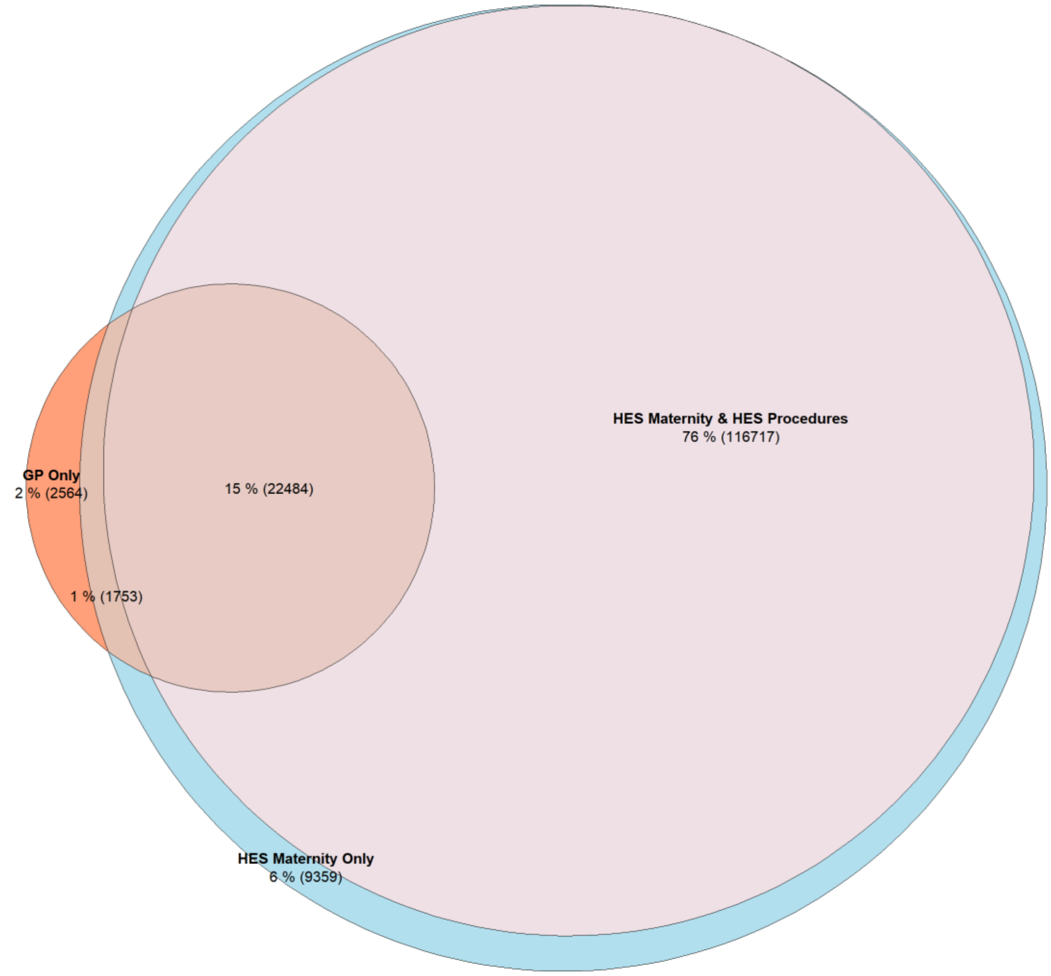

Legend: *Pregnancies suppressed by the Eulerr package due to low counts or omitted because of space restrictions: GP & HES Procedures (n=187). Pink circle = HES Procedures; blue circle = HES Maternity; orange circle = GP. Overlapping areas contain pregnancies with linking records in 1 or more datasets.

Supplementary Figure 7: Venn diagram – Evidence of linking delivery records (n=153,064)* across data sources ($\pm$7 days of delivery date) – 30 December 2020 to 4 February 2022.

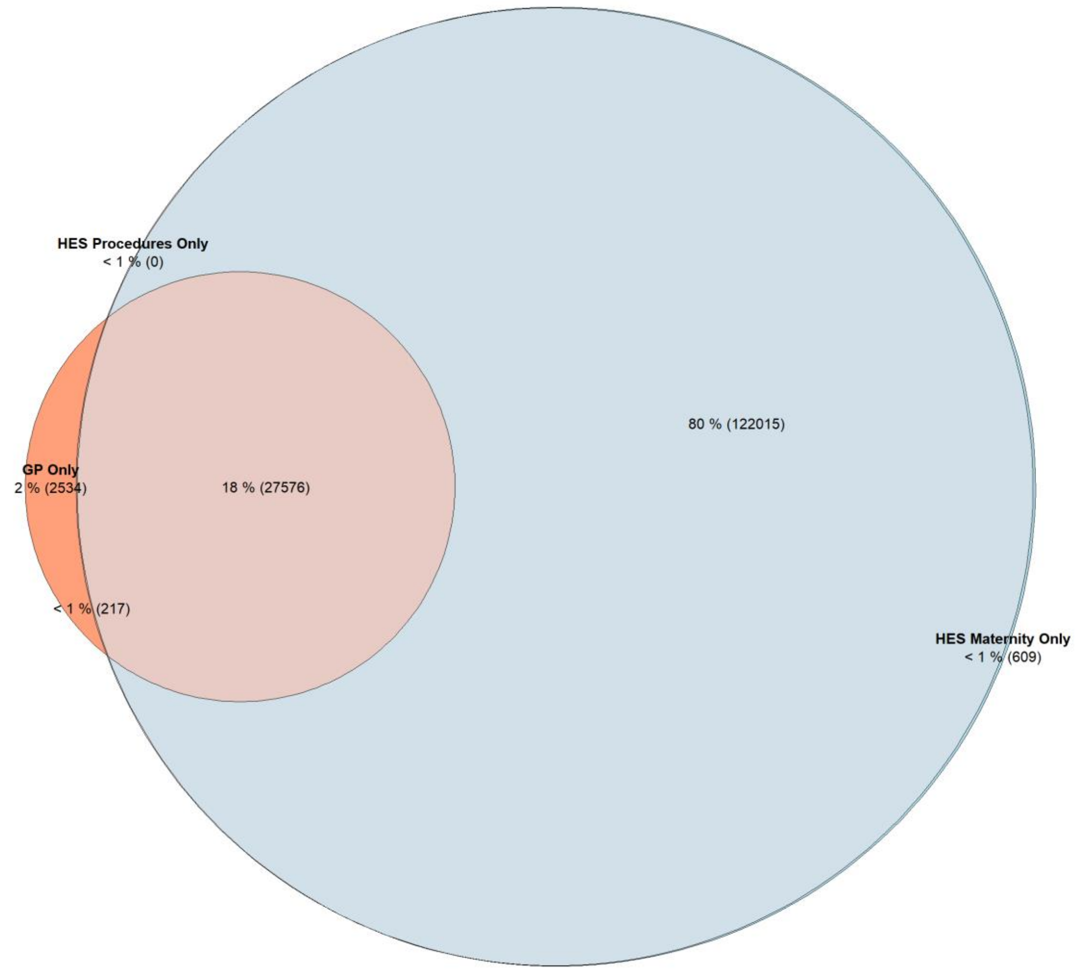

Legend: *Pregnancies suppressed by the Eulerr package due to low counts or omitted because of space restrictions: GP & HES Maternity (n=113). Pink circle = HES Procedures; blue circle = HES Maternity; orange circle = GP. Overlapping areas contain pregnancies with linking records in 1 or more datasets.

Supplementary Figure 8: Venn diagram - Evidence of linking pregnancy loss records (n=46,354)* across data sources (same day as loss date) – 30 December 2020 to 30 September 2022.

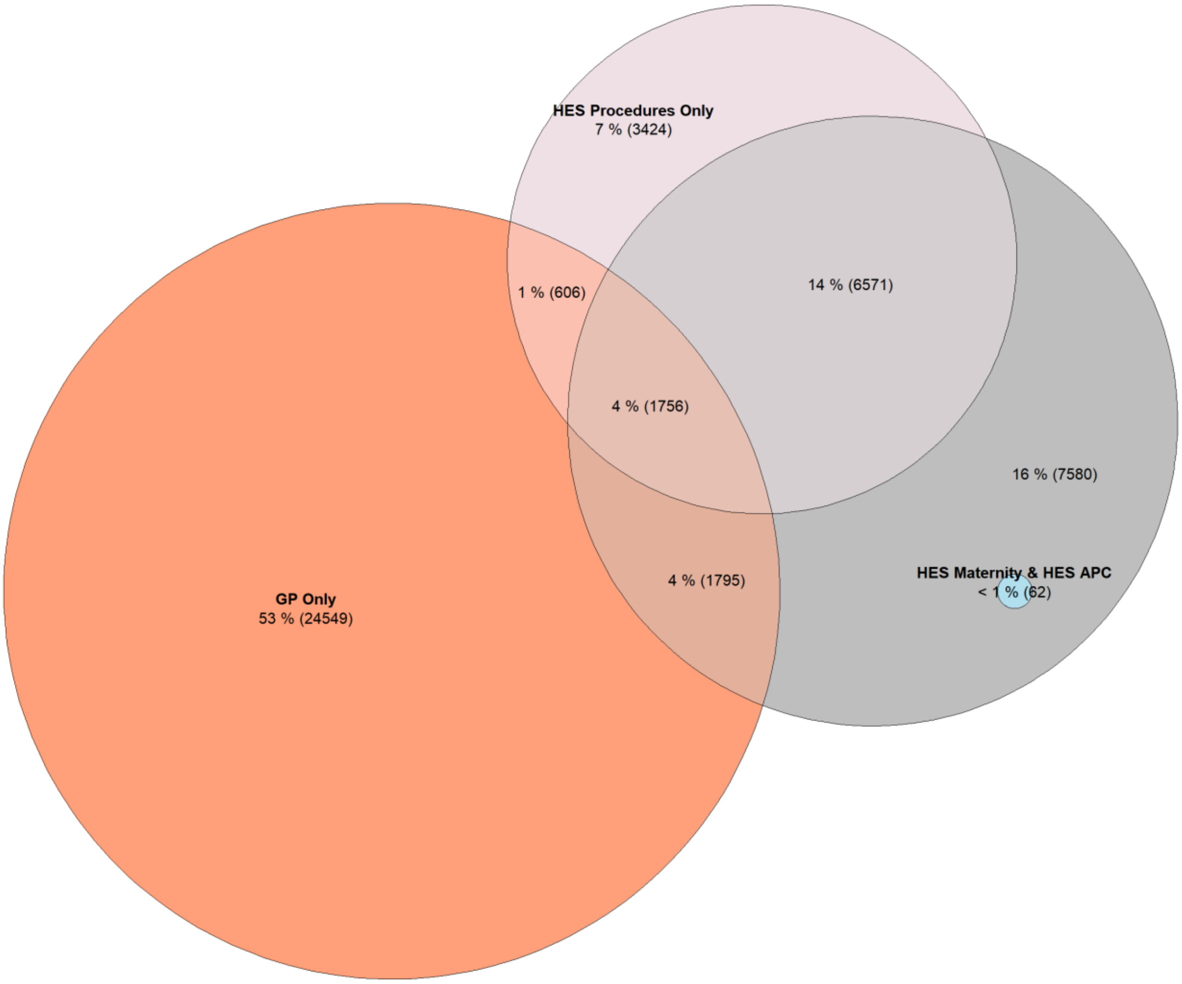

Legend: *Pregnancies suppressed by the Eulerr package due to low counts or omitted because of space restrictions: GP, HES Maternity, & HES APC (n=10); HES Maternity, HES Procedures, & HES APC (n=1). Orange circle = GP; pink circle = HES Procedures; blue circle = HES Maternity; dark grey circle = HES APC. Overlapping areas contain pregnancies with linking records in 1 or more datasets.

Supplementary Figure 9: Venn diagram - Evidence of linking pregnancy loss records (n=46,354)* across data sources ($\pm$7 days of loss date) – 30 December 2020 to 30 September 2022.

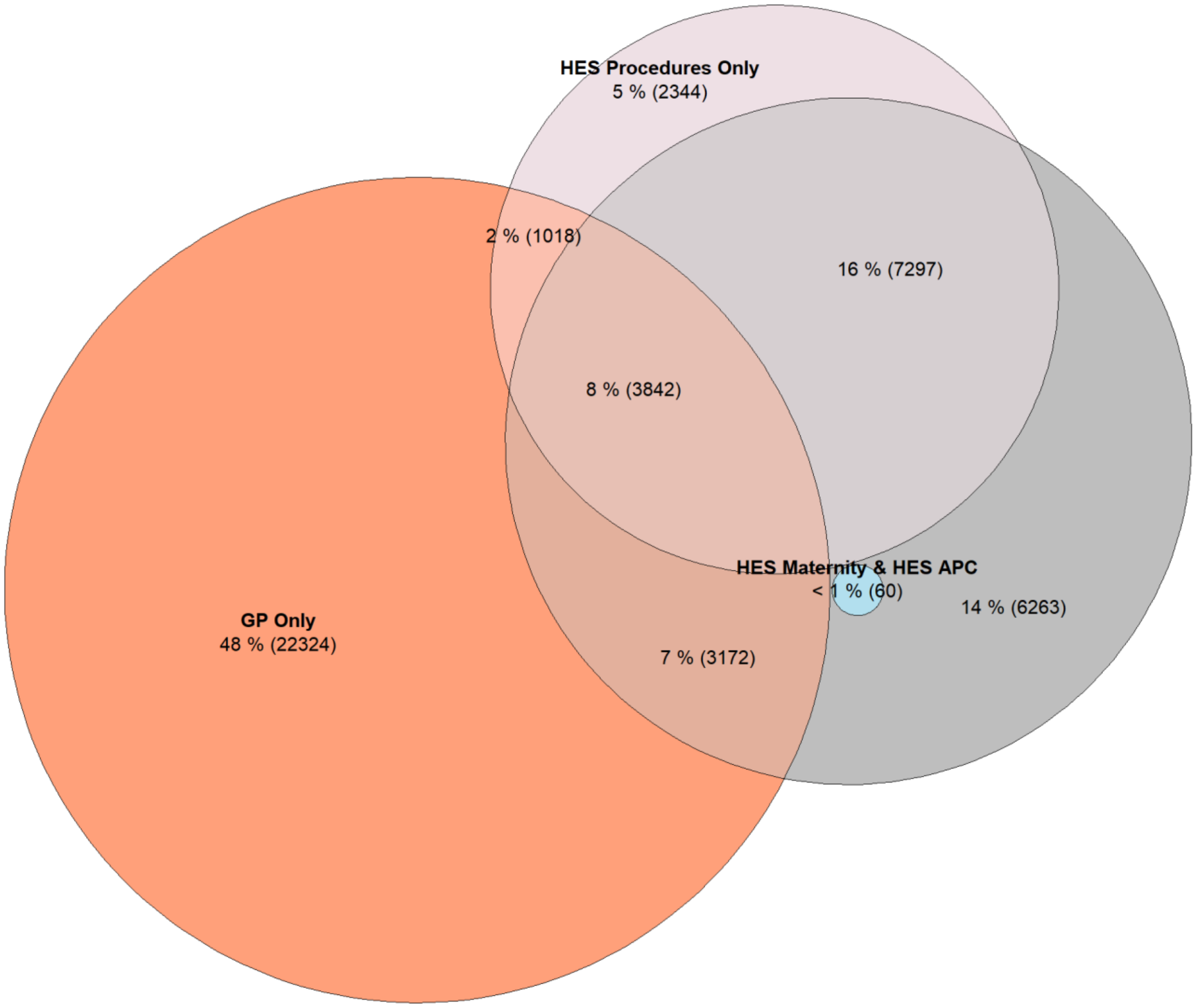

Legend: *Pregnancies suppressed by the Eulerr package due to low counts or omitted because of space restrictions: GP, HES Maternity, & HES APC (n=16); HES Maternity, HES Procedures, & HES APC (n=10); GP, HES Maternity, HES Procedures, & HES APC (n=8). Orange circle = GP; pink circle = HES Procedures; blue circle = HES Maternity; dark grey circle = HES APC. Overlapping areas contain pregnancies with linking records in 1 or more datasets.

Supplementary Figure 10: Venn diagram - Evidence of linking pregnancy loss records (n=37,594)* across data sources (same day as loss date) – 30 December 2020 to 4 February 2022.

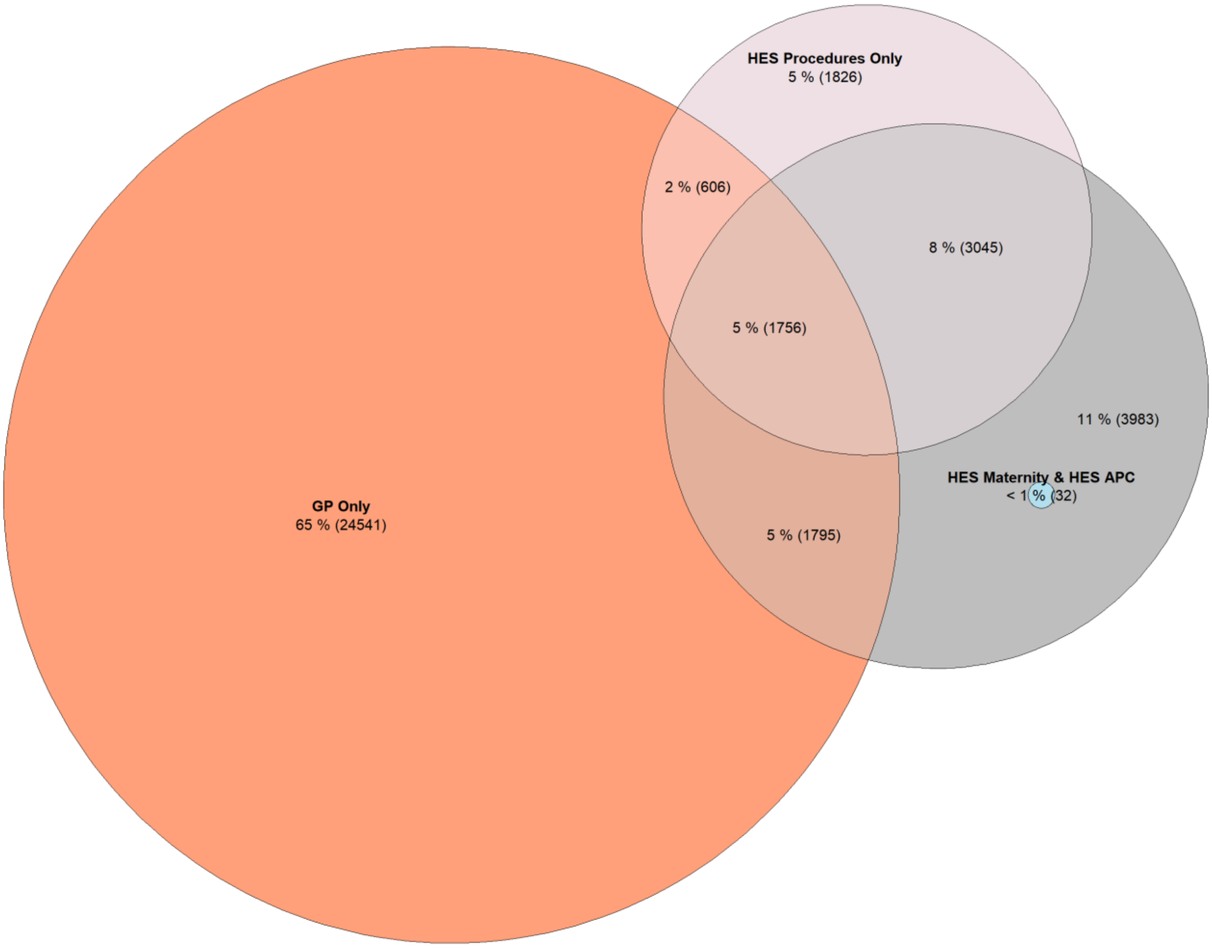

Legend: *Pregnancies suppressed by the Eulerr package due to low counts or omitted because of space restrictions: GP, HES Maternity, & HES APC (n=10). Orange circle = GP; pink circle = HES Procedures; blue circle = HES Maternity; dark grey circle = HES APC. Overlapping areas contain pregnancies with linking records in 1 or more datasets.

Supplementary Figure 11: Venn diagram - evidence of linking pregnancy loss records (n=37,594)* across data sources (±2 days of loss date) – 30 December 2020 to 4 February 2022.

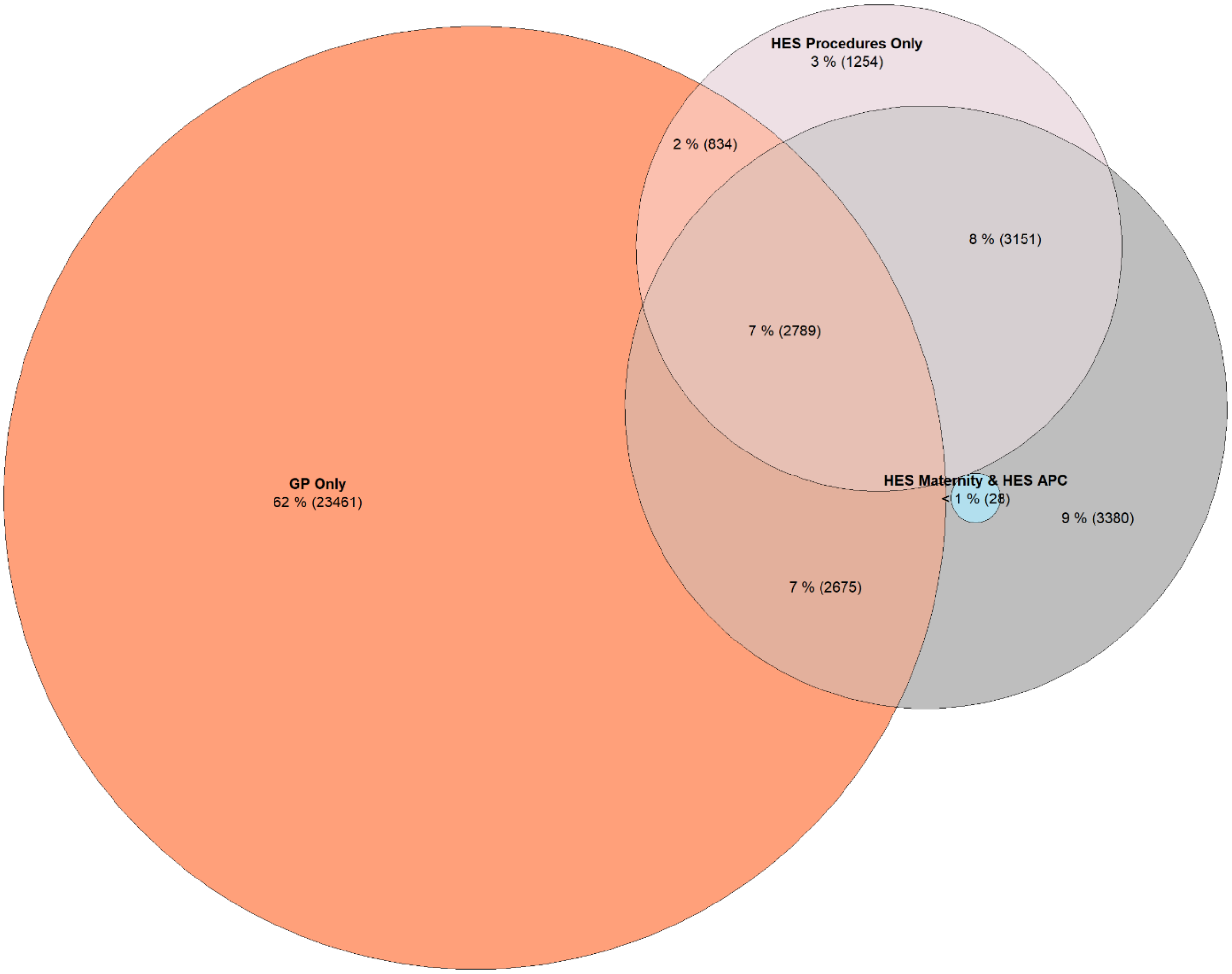

Legend: *Pregnancies suppressed by the Eulerr package due to low counts or omitted because of space restrictions: GP, HES Maternity, & HES APC (n=15); HES Maternity, HES Procedures, & HES APC (n=4); GP, HES Maternity, HES Procedures, & HES APC (n=3). Orange circle = GP; pink circle = HES Procedures; blue circle = HES Maternity; dark grey circle = HES APC. Overlapping areas contain pregnancies with linking records in 1 or more datasets.

Supplementary Figure 12: Venn diagram - Evidence of linking pregnancy loss records (n= 37,594)* across data sources (±7 days of loss date) – 30 December 2020 to 4 February 2022.

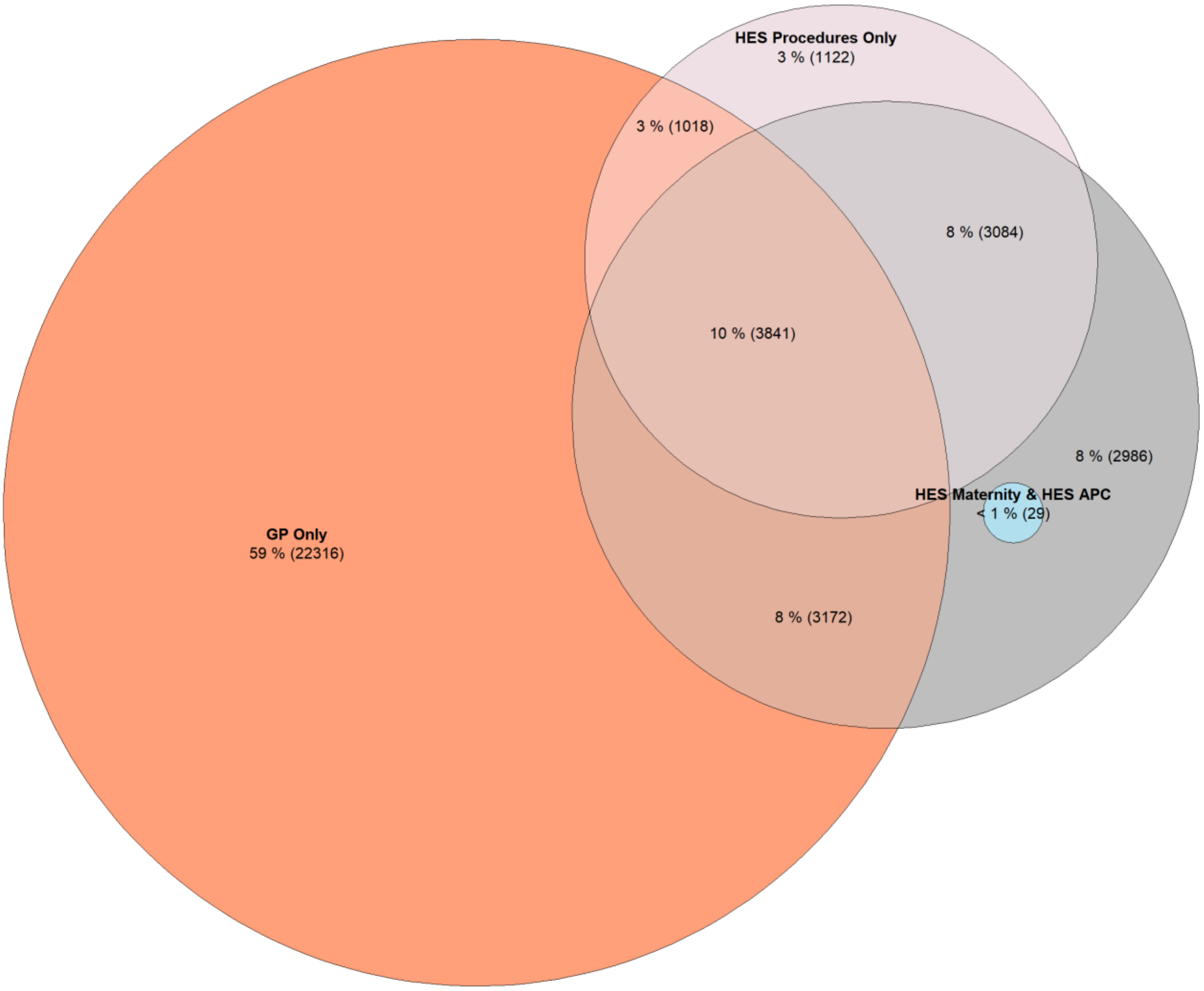

Legend: *Pregnancies suppressed by the Eulerr package due to low counts or omitted because of space restrictions: GP, HES Maternity, & HES APC (n=16); HES Maternity, HES Procedures, & HES APC (n=2); GP, HES Maternity, HES Procedures, & HES APC (n=8). Orange circle = GP; pink circle = HES Procedures; blue circle = HES Maternity; dark grey circle = HES APC. Overlapping areas contain pregnancies with linking records in 1 or more datasets.
