# Supplementary material for: Methods to establish a Pregnancy Register in the QResearch Database
Source: Commun Med (Lond). 2025 Dec 23;5:528. doi: 10.1038/s43856-025-01217-7 (PMC12727747; doi:10.1038/s43856-025-01217-7)
Supplement: Supplementary file 3 — Description of Supplementary files [file 43856_2025_1217_MOESM3_ESM.pdf]

Description of Supplementary files

- Supplementary Data 1 contains a list of the codegroups with SNOMED codes used to identify the outcomes, exposures, and covariates in GP data.
- Supplementary Data 2 contains a list of codegroups with ICD10 codes used to identify the outcomes, exposures, and covariates in hospital data.
- Supplementary Data 3 contains a list of codegroups with OPCS codes used to identify the outcomes, exposures, and covariates in hospital data.
- Supplementary Data 4 contains the tabulated demographic characteristics (age group, ethnicity, level of deprivation, and geographic region) of women who had one or more pregnancies during the COVID-19 Vaccination Programme (30 Dec 2020 - 11 February 2022).
- Supplementary Data 5 contains a tabulation of the demographic characteristics (age group, ethnicity, level of deprivation, and geographic region) of the cohort by the number of pregnancies during study period for the restricted cohort (pregnancy outcome between 30 December 2020 - 11 February 2022).
- Supplementary Data 6 contains a tabulation of the demographic characteristics (age group, ethnicity, level of deprivation, and geographic region) of the restricted cohort's (pregnancy outcome between 30 December 2020 - 11 February 2022) pregnancies by pregnancy outcome.
- Supplementary Data 7 contains a tabulation of the demographic characteristics (age group, ethnicity, level of deprivation, and geographic region) of restricted cohort (pregnancy outcome between 30 December 2020 - 11 February 2022) deliveries by outcome.
- Supplementary Data 8 contains the tabulated demographic characteristics (age group, ethnicity, level of deprivation, and geographic region) of restricted cohort (pregnancy outcome between 30 December 2020 - 11 February 2022) pregnancy losses by outcome during study period.
- Supplementary Data 9 contains a tabulation of pregnancy outcomes by data source for the restricted cohort (pregnancy outcome between 30 December 2020 – 11 February 2022).
- Supplementary Data 10: See Data Availability section at the end of the article file.
- Supplementary Data 11 contains the source data for Supp. Fig. 3, which is a Venn diagram of the number of delivery episodes with and without linking delivery (or delivery-related) records in one or more datasets on the same day as the delivery date between 30 December 2020 and 30 September 2022.
- Supplementary Data 12 contains the source data for Supp. Fig. 4, which is a Venn diagram of the number of delivery episodes with and without linking delivery (or delivery-related) records in one or more datasets within seven days of the delivery date between 30 December 2020 and 30 September 2022.
- Supplementary Data 13 contains the source data for Supp. Fig. 5, which is a Venn diagram of the number of delivery episodes with and without linking delivery (or delivery-related) records in one or more datasets on the same day as the delivery date between 30 December 2020 and 4 February 2022.
- Supplementary Data 14 contains the source data for Supp. Fig. 6, which is a Venn diagram of the number of delivery episodes with and without linking delivery (or delivery-related) records in one or more datasets within two days of the delivery date between 30 December 2020 and 4 February 2022.

- Supplementary Data 15 contains the source data for Supp. Fig. 7, which is a Venn diagram of the number of delivery episodes with and without linking delivery (or delivery-related) records in one or more datasets within seven days of the delivery date between 30 December 2020 and 4 February 2022.
- Supplementary Data 16: See Data Availability section at the end of the article file.
- Supplementary Data 17 contains the source data for Supp. Fig. 8, which is a Venn diagram of the number of pregnancy loss episodes with and without linking pregnancy loss records in one or more datasets on the same day as the pregnancy loss date between 30 December 2020 and 30 September 2022.
- Supplementary Data 18 contains the source data for Supp. Fig. 9, which is a Venn diagram of the number of pregnancy loss episodes with and without linking pregnancy loss records in one or more datasets within seven days of the pregnancy loss date between 30 December 2020 and 30 September 2022.
- Supplementary Data 19 contains the source data for Supp. Fig. 10, which is a Venn diagram of the number of pregnancy loss episodes with and without linking pregnancy loss records in one or more datasets on the same day as the pregnancy loss date between 30 December 2020 and 4 February 2022.
- Supplementary Data 20 contains the source data for Supp. Fig. 11, which is a Venn diagram of the number of pregnancy loss episodes with and without linking pregnancy loss records in one or more datasets within two days of the pregnancy loss date between 30 December 2020 and 4 February 2022.
- Supplementary Data 21 contains the source data for Supp. Fig. 12, which is a Venn diagram of the number of pregnancy loss episodes with and without linking pregnancy loss records in one or more datasets within seven days of the pregnancy loss date between 30 December 2020 and 4 February 2022.
